# Silver Nanoparticles Synthesized from *Enicostemma littorale* Exhibit Gut Tight Junction Restoration and Hepatoprotective Activity via Regulation of the Inflammatory Pathway

**DOI:** 10.3390/pharmaceutics17070895

**Published:** 2025-07-09

**Authors:** Hiral Aghara, Simran Samanta, Manali Patel, Prashsti Chadha, Divyesh Patel, Anamika Jha, Palash Mandal

**Affiliations:** P. D. Patel Institute of Applied Sciences, Charotar University of Science and Technology, Changa, Anand 388421, Gujarat, India; hiralaghara@gmail.com (H.A.); simran.samanta001@gmail.com (S.S.); pmanali6799@gmail.com (M.P.); prashstichadha@gmail.com (P.C.); divyesh2794@gmail.com (D.P.); anamikajha.bt@charusat.ac.in (A.J.)

**Keywords:** green synthesis, AgNPs, *Enicostemma littorale* dried leaves, hepatoprotective activity, gut barrier, alcohol-associated liver disease

## Abstract

**Background:** Alcohol-associated liver disease (ALD) is a primary global health concern, exacerbated by oxidative stress, inflammation, and gut barrier dysfunction. Conventional phytocompounds exhibit hepatoprotective potential but are hindered by low bioavailability. This study aimed to evaluate the hepatoprotective and gut-barrier-restorative effects of green-synthesized silver nanoparticles (AgNPs) derived from *Enicostemma littorale*, a medicinal plant known for its antioxidant and anti-inflammatory properties. **Methods**: AgNPs were synthesized using aqueous leaf extract of *E. littorale* and characterized using UV-Vis, XRD, FTIR, DLS, and SEM. HepG2 (liver) and Caco-2 (colon) cells were exposed to 0.2 M ethanol, AgNPs (1–100 µg/mL), or both, to simulate ethanol-induced toxicity. A range of in vitro assays was performed to assess cell viability, oxidative stress (H_2_DCFDA), nuclear and morphological integrity (DAPI and AO/EtBr staining), lipid accumulation (Oil Red O), and gene expression of pro- and anti-inflammatory, antioxidant, and tight-junction markers using RT-qPCR. **Results**: Ethanol exposure significantly increased ROS, lipid accumulation, and the expression of inflammatory genes, while decreasing antioxidant enzymes and tight-junction proteins. Green AgNPs at lower concentrations (1 and 10 µg/mL) restored cell viability, reduced ROS levels, preserved nuclear morphology, and downregulated CYP2E1 and SREBP expression. Notably, AgNPs improved the expression of Nrf2, HO-1, ZO-1, and IL-10, and reduced TNF-α and IL-6 expression in both cell lines, indicating protective effects on both liver and intestinal cells. **Conclusions**: Green-synthesized AgNPs from *E. littorale* exhibit potent hepatoprotective and gut-barrier-restoring effects through antioxidant, anti-inflammatory, and antilipidemic mechanisms. These findings support the therapeutic potential of plant-based nanoparticles in mitigating ethanol-induced gut–liver axis dysfunction.

## 1. Introduction

Over the past several years, plant-derived materials and natural compounds have been widely utilized for their anti-inflammatory and antioxidant properties in the treatment of various diseases. For example, curcumin has been employed as an anti-inflammatory agent in conditions such as atherosclerosis, arthritis, and inflammatory bowel disease [1]. In addition, other compounds such as silymarin, resveratrol, quercetin, and apigenin have demonstrated beneficial effects in a range of diseases, particularly as hepatoprotective agents [2]. Despite their therapeutic potential, a significant limitation of these plant-based compounds is their poor bioavailability, which greatly reduces their effectiveness. Oral and intestinal absorption of these compounds is typically low, ranging between 20–40% [3,4,5]. As a result, their protective efficacy at lower doses diminishes, often necessitating administration at higher concentrations, an approach that is not always feasible or safe.

To address this challenge, many researchers have proposed the green synthesis of nanoparticles as a promising strategy. Specifically, synthesizing nanoparticles from the same plant extracts that contain the bioactive natural compounds can significantly enhance their bioavailability. The green synthesis of metal nanoparticles using plant extracts has gained increasing attention in recent years due to its numerous advantages. These nanoparticles are generally more cost-effective to produce than chemically synthesized materials, and they exhibit improved absorption, a larger surface area, and reduced toxicity [6]. Additionally, green-synthesized nanoparticles incorporate plant-derived functional groups such as proteins, flavonoids, saponins, amino acids, and polyphenols that assist in both reducing metal ions and stabilizing nanoparticles [7,8]. Owing to their enhanced bioactivity, such nanoparticles are being explored as therapeutic agents for a wide range of diseases. Currently, a green-synthesized nanoparticle-mediated therapeutic approach for liver disease is being investigated [9].

Several hepatic diseases have been identified, including metabolic dysfunction-associated fatty liver disease (MAFLD), ALD, drug-induced liver injury, hepatic encephalopathy, and hepatitis. These conditions commonly impair liver metabolism and its regenerative capacity. Among the two major types of fatty liver disease, a drug targeting MAFLD has been approved by the Food and Drug Administration (FDA), whereas therapeutic options for ALD are still undergoing clinical evaluation. Silymarin, a key bioactive compound derived from *Silybum marianum*, is currently in Phase III clinical trials. Meanwhile, other treatments, such as probiotics and fecal microbiota transplantation, are also being assessed for their therapeutic potential. However, a significant limitation of silymarin is its poor water solubility, which leads to low bioavailability. To overcome this challenge, various nanoparticle-based approaches have been investigated for both MAFLD and ALD. Among these, formulations combining herbal compounds or green-synthesized nanoparticles have shown promising results in both in vitro and in vivo models [10,11]. In the case of ALD, chronic alcohol consumption leads to metabolic dysfunction, including the activation of cytochrome P450E1 (CYP2E1) and the overproduction of reactive oxygen species (ROS), thereby initiating an inflammatory cascade [12]. Furthermore, alcohol disrupts intestinal tight junctions, contributing to gut barrier dysfunction, which is often considered a secondary mechanism of liver injury. The resulting permeability allows toxic metabolites to translocate from the gut to the liver, exacerbating hepatic inflammation. These metabolites also induce immune responses that further harm gut epithelial cells. Therefore, an ideal therapeutic agent should be both non-toxic and anti-inflammatory to counteract the damage caused by ethanol. Given their advantageous characteristics, green-synthesized silver nanoparticles (AgNPs) may serve as effective agents in mitigating alcohol-induced damage to the gut–liver axis.

In this study, we used leaf extract from the Mamejavo plant (*Enicostemma littorale*), which is traditionally employed in the treatment of diabetes. This plant also exhibits potential anti-nephrotic activity, primarily attributed to the bioactive compound swertiamarin [13]. Given the therapeutic properties of both the plant extract and its active compound, their green synthesized AgNPs may serve as effective hepatoprotective agents. We hypothesize that AgNPs synthesized via green chemistry from *E. littorale* can help ameliorate ethanol-induced cellular damage. By restoring gut metabolites and reinforcing tight-junction integrity, these nanoparticles may prevent secondary liver injury and mitigate the progression of liver disease.

## 2. Materials and Methods

### 2.1. Materials

Silver nitrate was procured from Loba Chemicals (Mumbai, India). DAPI was obtained from Sigma-Aldrich (St. Louis, MO, USA), while H_2_DCFDA was purchased from Merck (Darmstadt, Germany). Acridine orange (AO) and ethidium bromide (EtBr) were sourced from SRL, and Oil Red O (ORO) powder was obtained from HiMedia (Mumbai, India). Reagents used for the gene expression analysis included RNAiso Plus (Cat. No. 9109) from Takara (Kusatsu, Japan), the Verso cDNA synthesis kit (Cat. No. AB1453) from Thermo Scientific, and PowerUp SYBR Green Master Mix from Thermo Scientific (Waltham, MA, USA). The HepG2, BRL3A, and Caco-2 cell lines were acquired from the National Centre for Cell Science (NCCS), Pune, India. MEM medium was purchased from HiMedia (Mumbai, India) and Gibco (Waltham, MA, USA). Trypsin-EDTA, the antibiotic-antimycotic solution, and fetal bovine serum (FBS) were all obtained from Gibco.

### 2.2. Methodology

#### 2.2.1. Synthesis of Green AgNPs from Plant Extract

The *Enicostemma littorale* Blume. plant was previously collected by Kirti Parwani. The plant was collected during the monsoon season from the Saurashtra region of Gujarat, India. The plant was further dried and ground into powder using a crusher [13]. For the preparation of the plant extract, 2.5 g of dried powdered leaves of *Enicostemma littorale* were used. Dried powder leaves were further added to 100 mL of sterile distilled water and stirred on a magnetic hot plate stirrer for 30 min at 60 °C and 250 RPM. After stirring on a magnetic stirrer, the water extract was filtered through Whatman No. 1 filter paper (Marlborough, UK). The extract was further filter sterilized in a clean, sterile tube using a 0.2 µm syringe filter. The sterile water extract was stored at 4 °C for future use [14].

For the synthesis of AgNPs, 2 mL of the aqueous plant extract was mixed with 60 mL of a 1 mM silver nitrate (AgNO_3_) solution and incubated at 50 °C for 6 h. The entire process was carried out in a dark room to prevent any photoactivation of AgNO_3_. The initiation of nanoparticle formation was indicated by a visible color change in the solution, from pale yellow to dark brown, upon the completion of incubation. To separate the synthesized nanoparticles, the reaction mixture was centrifuged at 15,000× *g* for 20 min. The supernatant was discarded, and the resulting pellet, containing the AgNPs, was collected. Finally, the pellet was air-dried to obtain dry nanoparticle powder, which was then used for further characterization and experimental procedures.

For experimental purposes, AgNPs were weighed and suspended in Milli-Q water, which was then further filter sterilized in a new sterile tube using a 0.2 µm syringe filter. The suspension was further bath sonicated for 20 to 30 min prior to the experiments. This facilitated the proper dispersion of nanoparticles in culture media.

#### 2.2.2. Determination of AgNPs

Prior to characterization, the synthesized silver nanoparticles (AgNPs) were suspended in distilled water and sonicated in a bath sonicator for 30 min to ensure uniform dispersion. Optical properties were assessed using a UV–Visible spectrophotometer (Shimadzu UV-1800, Kyoto, Japan) in the spectral range of 300–700 nm. Distilled water was used as the reference solvent since the AgNPs were dispersed in it. The absorbance spectrum was used to evaluate the quality and confirm the synthesis of AgNPs from the plant extract.

To determine the hydrodynamic diameter and polydispersity index (PDI), dynamic light scattering (DLS) analysis was performed at a scattering angle of 90°, based on the principle of Brownian motion. The crystalline nature of the nanoparticles was analyzed via X-ray diffraction (XRD) in the 2θ range of 20° to 80°, using Ni-filtered Cu Kα radiation (λ = 1.5 Å) in accordance with Bragg’s law, where 2θ denotes the angle between the incident and reflected X-rays (XRD, Bruker, Billerica, MA, USA, D2 Phaser).

Fourier-transform infrared spectroscopy (FTIR) was employed to identify functional groups involved in the reduction and stabilization (capping) of AgNPs. The conventional potassium bromide (KBr) pellet method was used for sample preparation. An aqueous solution of AgNPs was mixed with 100 mg of KBr and left to dry overnight. The next day, a thin film was prepared using 9 mg of KBr and AgNPs containing KBr in a 9:1 ratio. The thin film was further evaluated using a SHIMADZU IRSpirit-X FTIR spectrometer with a wavelength range of 400 cm^−1^ to 4000 cm^−1^; it was scanned 45 times. For the morphological analysis, the nanoparticle suspension was drop-cast onto a coverslip and imaged using scanning electron microscopy (SEM) (Jeol, JSM- 6010LA, Tokyo, Japan) and FE-SEM (Thermo Fisher, Phenom Pharos G2, Waltham, MA, USA) to observe the shape and surface structure of the silver nanoparticles [14].

#### 2.2.3. Cell Culture Studies

For cell culture experiments, human-derived HepG2 (hepatocellular carcinoma), BRL3A (rat liver normal fibroblast), and Caco-2 (colorectal adenocarcinoma) cell lines were obtained from the National Centre for Cell Science (NCCS), Pune. HepG2 and BRL3A cells were cultured in Minimum Essential Medium (MEM) supplemented with 10% fetal bovine serum (FBS) and 1× non-essential amino acids. Caco-2 cells were maintained in low-glucose Dulbecco’s Modified Eagle Medium (DMEM) supplemented with 20% FBS and 1× antibiotic-antimycotic solution containing penicillin, streptomycin, and amphotericin B. The cell passage numbers between 35 and 45 were used for experimental purposes for cancerous cells, while the viability experiment on BRL3A was carried out from passage numbers 20–23. For the experimental treatments, cells were incubated in low-serum media. All cell lines were maintained at 37 °C in a humidified incubator with 5% CO_2_.

##### Cell Viability Study via 3-(4,5-Dimethylthiazol-2-yl)-2,5-diphenyltetrazolium Bromide (MTT)

The cytotoxicity of AgNPs was evaluated using the MTT assay on HepG2, BRL3A, and Caco-2 cell lines. Approximately 11,000–15,000 Caco-2 cells and 20,000–25,000 HepG2 and BRL3A cells were seeded into 96-well plates individually and incubated for 24 h. Once the cells reached 45–50% confluency, they were treated with varying concentrations of AgNPs (1–100 µg/mL), both alone and in combination with 0.2 M ethanol (prepared in a culture medium). The final volume in each well was maintained at 100 µL.

After 24 h of treatment, cells were washed with PBS and incubated with a fresh medium containing MTT reagent at a final concentration of 0.5 mg/mL. The plates were incubated at 37 °C in the dark for 1 to 4 h. Following incubation, the MTT-containing medium was removed, and formazan crystals were solubilized using 100% DMSO. Absorbance was measured at 570 nm using a multimode ELISA plate reader, and cell viability was calculated using the following equation [15]:

Cell viability %=AsAc×100

where *As* = absorbance of test and *Ac* = absorbance of control.

#### 2.2.4. Cell Damage Study

##### AO/EtBr Dual Staining

Acridine orange (AO) and ethidium bromide (EtBr) dual staining was employed to assess cell death, nuclear morphological changes, and the formation of apoptotic bodies. The same number of HepG2 and Caco-2 cells as used in previous assays were seeded into 96-well plates. Once the cells reached 40–50% confluency, they were treated with 0.2 M ethanol, AgNPs alone, or a combination of ethanol and AgNPs. After 24 h of treatment, the cells were washed with phosphate-buffered saline (PBS) and stained with a 100 µg/mL AO/EB solution. The stained cells were immediately visualized under a fluorescence inverted microscope (Nikon Eclipse 3000, Tokyo, Japan) using FITC and TRITC filters. Live cells with intact membranes appeared green under merged channel imaging. Cells showing chromatin condensation (dense green nuclei) indicated early apoptosis, while those with compromised membrane integrity fluoresced red, signifying late apoptosis or necrotic cell death [16].

##### DAPI Staining for Nuclear Morphology Study

HepG2 and Caco-2 cells were seeded into 96-well plates at a density of 20,000–25,000 and 11,000–15,000 cells per well, respectively. After reaching the appropriate confluency, the cells were treated with 0.2 M ethanol and 1 µg/mL of AgNPs, followed by incubation for 24 h. After treatment, the cells were gently washed with 1X phosphate-buffered saline (PBS) and fixed using 4% formaldehyde. Residual fixative was removed in subsequent washes with distilled water and PBS. The cells were then stained with 100 µM DAPI in the dark for 15 min. Following staining, excess DAPI was removed by washing with PBS. Fluorescence imaging was performed using a fluorescence microscope, and the emission intensity was quantified using a multimode reader (PerkinElmer, Waltham, MA, USA) at an excitation wavelength of 385 nm. Fold change in fluorescence intensity was calculated relative to untreated control cells [15].

##### Reactive Oxygen Species (ROS) Estimation

ROS levels were measured using the fluorescent probe 2′,7′-dichlorodihydrofluorescein diacetate (H_2_DCFDA). HepG2 and Caco-2 cells were seeded in 96-well plates at the same densities as previously described. After reaching the appropriate confluency, cells were treated with 0.2 M ethanol and 1 µg/mL of AgNPs and incubated for 24 h. Following treatment, the cells were washed with 1×PBS and fixed with 4% formaldehyde. Residual fixative was removed with additional PBS washes. The cells were then incubated with 20 µM H_2_DCFDA in the dark for 1 h. After staining, excess dye was removed by washing with PBS. Fluorescence intensity was recorded using a microplate reader (Perkin Elmer, Waltham, MA, USA) at an excitation/emission wavelength of 480/530 nm [15,17] and fold change was calculated against normal untreated control cells.

##### Lipid Accumulation Estimation

Ethanol exposure can induce intracellular lipid accumulation, which can be detected using lipophilic dyes such as ORO. To quantify lipid content, HepG2 and Caco-2 cells were seeded in 96-well plates at the previously mentioned densities and incubated until they reached 40–50% confluency. The cells were then treated with 0.2 M ethanol, AgNPs (1 µg/mL), and their combination to evaluate the protective effect of AgNPs against ethanol-induced lipid accumulation. After 24 h of treatment, cells were stained with ORO following the protocol established in our previous study [15]. For the quantitative analysis of lipid content, the retained stain within the cells was extracted using 100% isopropanol (IPA), and the absorbance was measured to determine total lipid accumulation.

#### 2.2.5. Gene Expression

##### RNA Isolation

As ethanol damages cells and increases the levels of proinflammatory cytokines while decreasing the levels of anti-inflammatory cytokines, this can be further estimated through gene expression analysis. To this end, 6-well plates were used, and, in those plates, HepG2 cells were seeded at approximately 7–8 × 10^6^ and Caco-2 cells were seeded at approximately 5–6 × 10^6^. As the cells reached the proper confluency, they were treated with ethanol and AgNPs and incubated for 24 h. After the incubation period was completed, the cells were exposed to the cell lysis solution. Here, we used RNA isoplus from Takara for cell lysis. Briefly, 1 mL of RNAiso Plus was added to each well and incubated at room temperature for 2 min to lyse the cells. The lysate was then transferred to microcentrifuge tubes, followed by the addition of chloroform. After vigorous mixing, the samples were centrifuged at 12,000× *g* for 15 min at 4 °C. After the centrifugation, the top transparent layer was collected in a clean microcentrifuge tube and further combined with 100% IPA. These tubes were further incubated at RT for 10 min and then again centrifuged at 12,000× *g* for 10 min at 4 °C. At the bottom, a pellet appeared after centrifugation, which was further washed with 75% ethanol and centrifuged at 7500× *g* for 5 min at 4 °C. The supernatant was discarded, and the pellet was resuspended in nuclease-free sterile water. The pure total RNA can be stored at −80 °C for future use and better stability.

##### cDNA Synthesis

For the cDNA synthesis process, the total RNA concentration was checked using a Nanodrop (Nanodrop 2000, Thermo Fisher). The ratios of 260/280 and 260/230 should be close to 2.0 for pure RNA. Further cDNA was synthesized using the Verso cDNA synthesis kit from Thermo Scientific. Here, 1 µg of total RNA was used to synthesize cDNA.

##### qPCR for Gene Expression Analysis

Quantitative real-time PCR (qPCR) was performed using 5 ng of synthesized cDNA as the template in a total reaction volume of 10 μL. Each reaction contained 500 nM of both forward and reverse primers, as well as SYBR Green master mix. The thermal cycling conditions were initiated with a hold at 95 °C for enzyme activation, followed by 40 cycles of denaturation at 95 °C for 30 s, annealing at 60 °C for 45 s, and extension at 72 °C for 1 min. Relative gene expression was calculated using the 2^ΔΔCt^ method. 18S rRNA was used as the internal housekeeping control. Gene expression analysis was conducted in both HepG2 and Caco-2 cell lines. The list of primer sequences used is provided in Table 1.

### 2.3. Statistical Analysis

All experiments were conducted independently at least three times to ensure reproducibility and reliability. Data were analyzed using GraphPad Prism software version 9.0. Statistical analysis was performed using one-way or two-way ANOVA, followed by appropriate multiple comparison tests, depending on the experimental design. Results are expressed as the mean ± standard deviation (SD) from three to six individual replicates.

## 3. Results

### 3.1. Synthesis and Characterisation of AgNPs

#### 3.1.1. Synthesis of Nanoparticles

**Figure 1:**
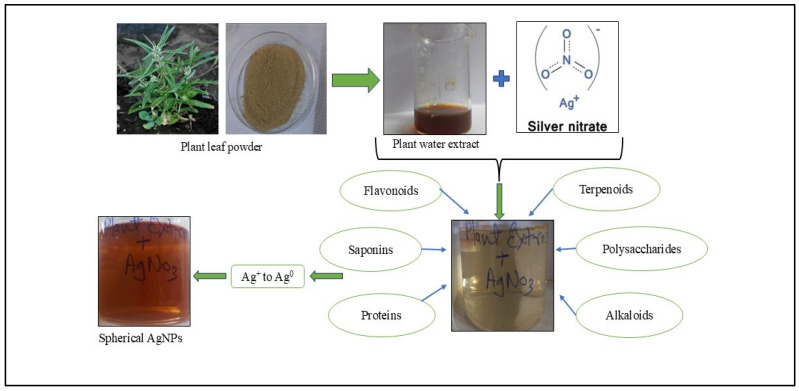
The plant dried leaf powder was suspended in water and stirred for a few minutes at a high temperature to ensure the compound was present in the water extract. Further combining this with a silver nitrate solution resulted in the formation of nanoparticles in the presence of biomolecules in the solution. The color change in the solution after the incubation period indicates the formation of nanoparticles.

The green synthesis method was used for the formation of AgNPs. The synthesis process was visually confirmed by a distinct color change from light yellow to brown (Figure 1), indicating the reduction of Ag^+^ ions to metallic Ag^0^, a characteristic property of silver nanoparticles. This reduction is facilitated by the bioactive compounds present in the plant extract.

#### 3.1.2. Characterization of AgNPs

**Figure 2:**
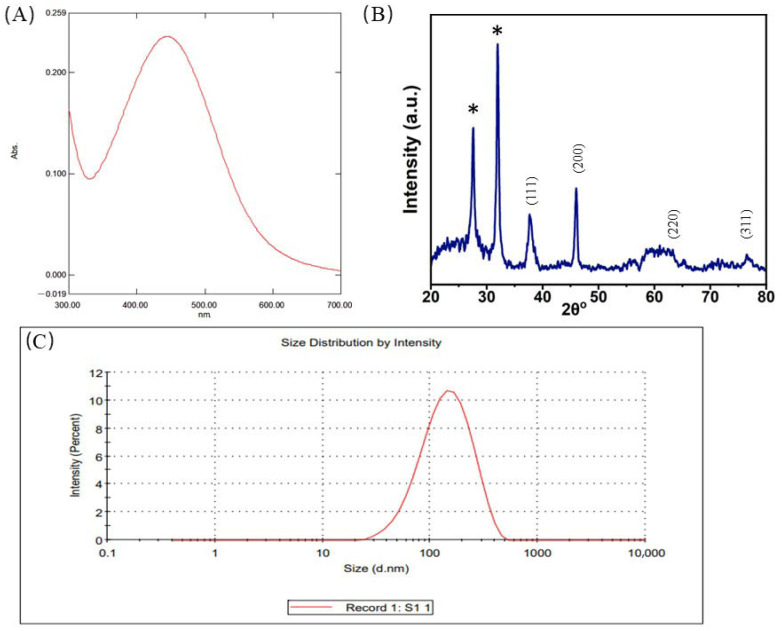
Characterization of AgNPs synthesized from plant leaf extract. (**A**) UV–Visible spectroscopy of green AgNPs synthesized from plant extract showing a prominent peak at 445 nm. (**B**) X-ray diffraction pattern of synthesized AgNPs (* extra peaks generated due to the plant extract.). (**C**) Size distribution of green AgNPs.

The color change of the suspension from pale yellow to dark brown indicates the visual confirmation of AgNPs formation. To further validate this, UV–Visible spectroscopy was employed to analyze the optical properties of the nanoparticles in aqueous solution. The absorbance spectra were recorded in the range of 300–700 nm. A distinct peak was observed at 445 nm (Figure 2A).

The diffractogram illustrating the characteristics of AgNPs was obtained through X-ray diffraction (XRD) analysis to validate the crystalline structure of the AgNPs. The resultant spectrum revealed prominent peaks observed at 2θ = 37.98°, 46.08°, 64.69°, and 76.54°, corresponding to the Bragg reflections indicative of the face cantered cubic arrangement of silver lattice planes, specifically the (111), (200), (220), and (311) planes (Figure 2B).

The hydrodynamic size and polydispersity index of the AgNPs in suspension were determined using dynamic light scattering (DLS). The peak size is 155.8 d.nm, and the z-average size was 119.5 d.nm (Figure 2C). A polydispersity index (PdI) lower than 0.7 indicates that the sample is monodispersed in nature [18].

**Figure 3:**
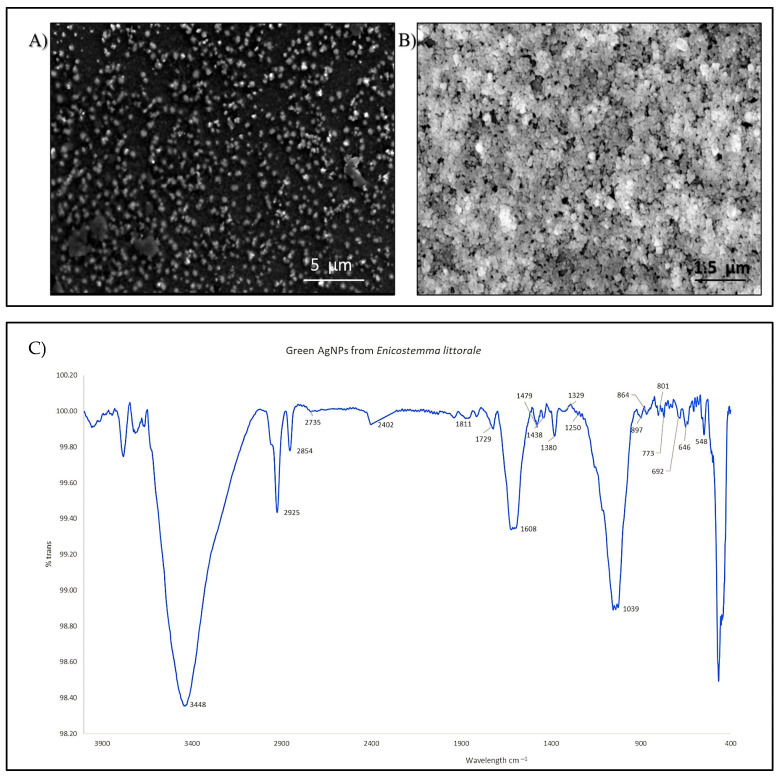
Characterization of AgNPs. (**A**) shows the SEM image of AgNPs at 5 µm resolution. (**B**) FE-SEM image of green AgNPs at 1.5 µm resolution. (**C**) FTIR analysis of green AgNPs showing the peaks.

The FTIR plot shows the peaks at 3448, 2924, 2854, and 2735 cm^−1^. Furthermore, the peaks at 1729, 1608, and 1438 cm^−1^ correspond to the peak of the extracted component. Peaks at 1380, 1329, 1250, and 1039 cm^−1^ correspond to the presence of alkaloids in the extract compound. The peak from 850 to 600 cm^−1^ is related to other organic compounds present in the extract (Figure 3C). The peak at 548 cm^−1^ is due to the presence of Ag-O [19]. Further bonds are discussed in Section 4 and along with Table 2.

The morphology of the AgNPs with *Enicostemma littorale* was determined with the help of SEM. Here, the AgNPs demonstrate uniformity in shape, which is spherical. The results from another research paper that synthesizes AgNPs with plant extracts are almost identical to those in this study, showing a similar spherical morphology (Figure 3A,B).

### 3.2. Cell Culture

#### 3.2.1. Cell Viability Study

**Figure 4:**
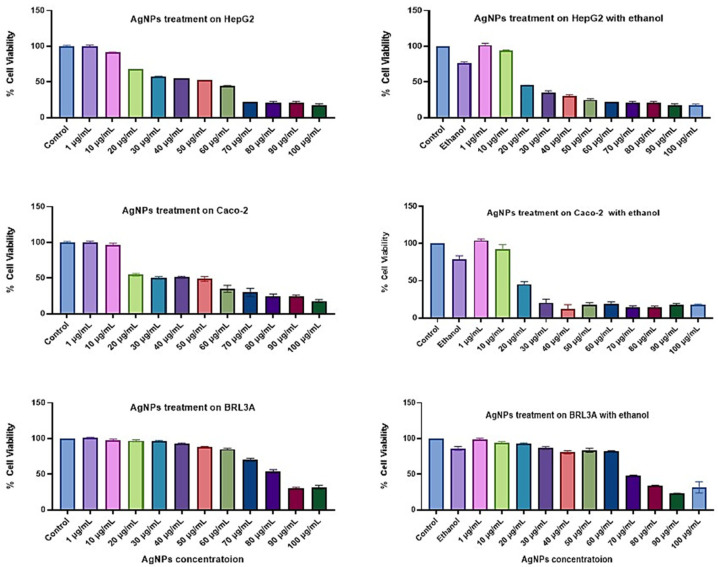
Cell viability study of green AgNPs on cell lines, HepG2, Caco-2, and BRL3A. Cell viability was studied for AgNPs alone and in combination with 0.2 M ethanol. Statistical analysis: data are presented as the mean ± SD (*n* = 3).

Green-synthesized AgNPs were initially evaluated for their cytotoxicity. Cells were treated with AgNPs alone for 24 h, as well as in combination with 0.2 M ethanol. Comparable cell viability was observed in both conditions. When treated with AgNPs alone, a gradual decline in viability was noted beyond 10 µg/mL, with a sharp drop observed at 20 µg/mL. In ethanol-treated cells, viability remained at approximately 75% in HepG2 and 82% in Caco-2 cells. Interestingly, co-treatment with AgNPs and ethanol resulted in increased cell viability at concentrations of 1 and 10 µg/mL, reaching up to 100%, suggesting the protective effect of AgNPs against ethanol-induced cytotoxicity. However, at concentrations above 10 µg/mL, cell viability significantly dropped to around 20% in both conditions, indicating the potential toxicity of AgNPs at higher doses (Figure 4).

In contrast, BRL3A cells did not exhibit significant cell death even at higher concentrations of AgNPs alone. However, cells co-treated with ethanol and AgNPs exhibited substantial cell death after the treatment with 60 µg/mL AgNPs. In conclusion, normal cells treated with 0.2 M ethanol showed nearly 14% cell death, which was mitigated when treated with the lowest concentration of AgNPs.

#### 3.2.2. Cell Damage Study

##### AO/EtBr Dual Staining

**Figure 5:**
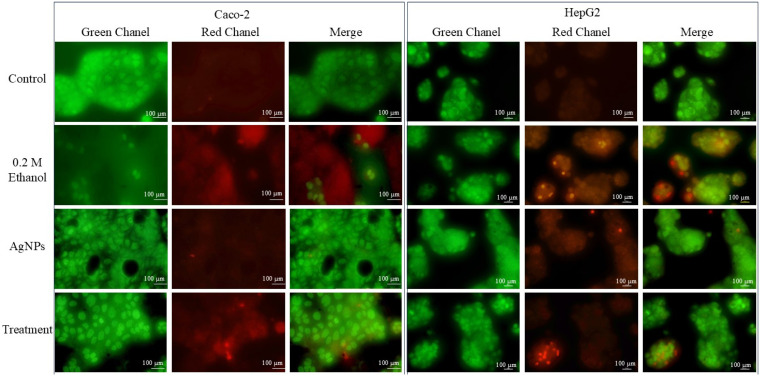
AO/EtBr staining of HepG2 and Caco-2 cells. In both cell types, ethanol-treated cells showed more damage, whereas, in the treatment group, less damage was observed. The image was taken with a fluorescent microscope at a resolution of 100 µm. The experiment was carried out in triplicate.

The dual staining technique provided clear visual evidence of ethanol-induced cellular damage and death. In contrast, cells treated with lower concentrations of AgNPs alone maintained intact morphology, indicating minimal harm. When cells were co-treated with AgNPs and ethanol, the damage caused by ethanol was significantly reduced, and signs of cell proliferation were observed. As shown in Figure 5, both cell lines exhibited substantial damage upon exposure to ethanol. However, this effect was notably reversed in the co-treatment group. Quantitative analysis revealed that approximately 20 ± 3 out of 100 cells were damaged in the ethanol-treated group, whereas only about 10 ± 2 cells were affected in the co-treated group, demonstrating a significant reduction in cellular damage. The double staining method employed acridine orange and ethidium bromide, two fluorescent DNA-binding dyes that differentiate between live and damaged cells. Ethanol-compromised cells, having lost membrane integrity, absorbed the red dye, resulting in red-stained nuclei. In contrast, co-treatment with AgNPs significantly reduced the number of red-stained cells, further confirming the protective effect of AgNPs against ethanol-induced cytotoxicity.

##### DAPI Staining for Nuclear Morphology

**Figure 6:**
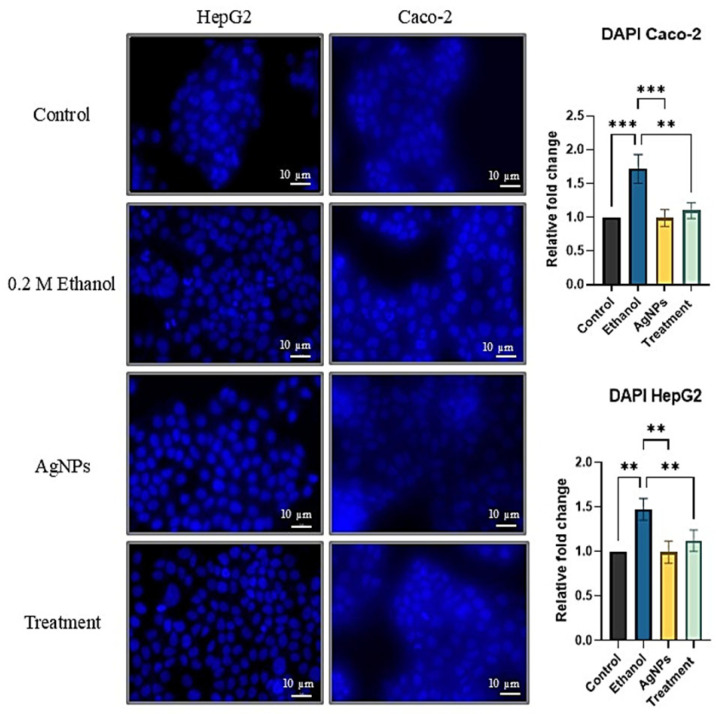
DAPI staining shows that ethanol exposure caused nuclear damage in HepG2 and Caco-2 cells, while AgNPs preserved nuclear integrity. Co-treatment with AgNPs reduced ethanol-induced damage, as reflected by restored nuclear morphology and decreased DAPI fluorescence intensity. Statistical analysis: one-way ANOVA with Dunnett’s post hoc test; compared to the ethanol group, where ** *p* < 0.01, *** *p* < 0.001.

Due to the presence of ethanol, cells exhibited distorted nuclear morphology, as observed in Figure 6. Ethanol-treated cells displayed clear signs of apoptosis, including nuclear fragmentation and the presence of apoptotic bodies, indicating ethanol-induced cellular damage. In contrast, cells treated with 1 µg/mL of AgNPs alone maintained normal nuclear morphology, comparable to that of untreated control cells. In the treatment group, where cells were exposed to both ethanol and AgNPs, the nuclei remained largely intact, with only a few cells exhibiting nuclear distortion. Furthermore, analysis of DAPI fluorescence intensity revealed that treated cells exhibited reduced fluorescence, suggesting preserved nuclear integrity and reduced DNA damage.

##### ROS Estimation

**Figure 7:**
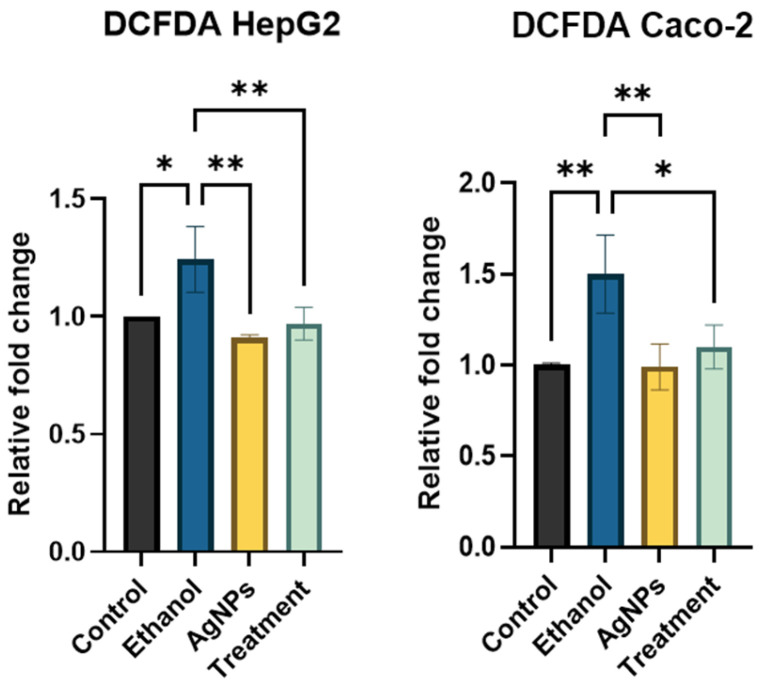
Quantitative ROS estimation showing a higher relative ratio in ethanol-treated cells, while the ratio decreased in the treatment group. Statistical analysis: one-way ANOVA with Dunnett’s post hoc test; compared to the ethanol group, where ** *p* < 0.01, * *p* < 0.05.

Intracellular ROS levels were assessed using the fluorescent probe H_2_DCFDA. Ethanol-treated cells exhibited a marked increase in fluorescence intensity relative to the control group, indicating elevated ROS production. ROS oxidizes H_2_DCFDA to form the fluorescent compound 2′,7′-dichlorofluorescein, thereby serving as an indicator of oxidative stress. Cells exposed to AgNPs alone showed no significant change in fluorescence, suggesting that the nanoparticles do not induce oxidative stress. Notably, co-treatment with ethanol and AgNPs resulted in a reduced fluorescence intensity, indicating that AgNPs effectively attenuate ethanol-induced ROS generation (Figure 7).

##### Lipid Accumulation Study

**Figure 8:**
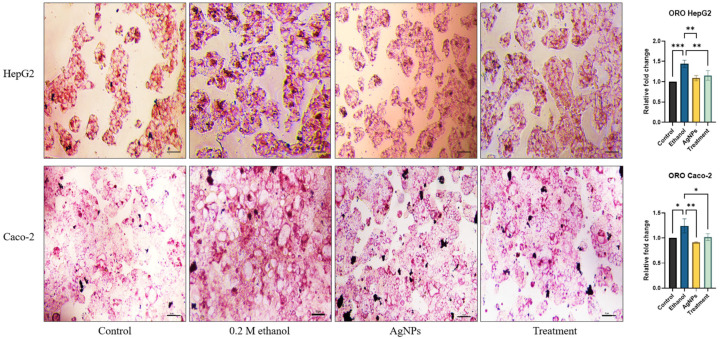
ORO staining to assess intracellular lipid accumulation in HepG2 and Caco-2 cells. Cells were treated with 0.2 M ethanol, AgNPs (1 µg/mL), or a combination of both (treatment) and compared to untreated controls. Ethanol-treated cells exhibited significant lipid accumulation, evident as dense, red-stained droplets in the cytoplasm. AgNPs alone did not alter lipid levels compared to the control, while co-treatment with AgNPs reduced ethanol-induced lipid accumulation. The image was taken in a bright field microscope with a resolution of 10 µM. Statistical analysis: data are presented as the mean ± SD, one-way ANOVA with Dunnett’s post hoc test; compared to the ethanol group, where * *p* <0.05, **p < 0.01, *** *p* < 0.001.

As shown in Figure 8, ethanol-treated HepG2 and Caco-2 cells exhibited a marked increase in intracellular lipid accumulation compared to the control group, with a fold change of approximately 0.7. Cells treated with AgNPs alone displayed lipid levels comparable to those of the control, as determined by both microscopic visualization and quantitative analysis, indicating no adverse effect on lipid metabolism. In the co-treatment group, lipid accumulation was notably reduced relative to the ethanol-only group, suggesting that AgNPs effectively mitigate ethanol-induced lipid buildup and potentially contribute to improved cell viability.

##### Gene Expression Study

**Figure 9:**
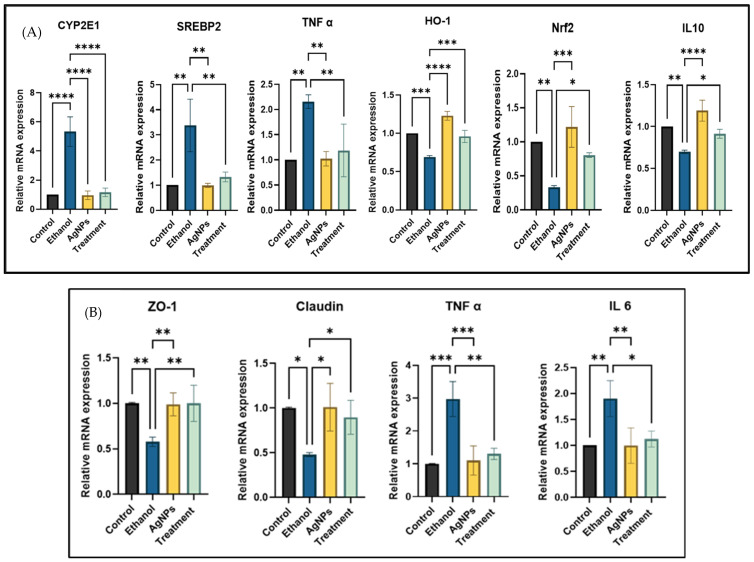
Gene expression analysis in HepG2 and Caco-2 cells with and without ethanol exposure. (**A**) HepG2 cells: relative mRNA expression of CYP2E1, SREBP2, pro-inflammatory (e.g., TNF-α, IL-6), anti-inflammatory (e.g., IL-10), and antioxidant (Nrf2, HO-1) genes. (**B**) Caco-2 cells: gene expression of tight-junction markers (ZO-1, claudin) and pro-inflammatory cytokines. Data are presented as mean ± SD (*n* = 3); statistical analysis was performed using one-way ANOVA with Dunnett’s post hoc test. Comparisons were made against the ethanol-treated group, where * *p* < 0.05, ** *p* < 0.01, *** *p* < 0.001, and **** *p* < 0.0001.

Ethanol exposure in HepG2 cells resulted in a significant upregulation of CYP2E1 expression, showing nearly a fivefold increase compared to control cells. However, co-treatment with AgNPs markedly reduced CYP2E1 expression levels. As CYP2E1 plays a key role in generating ROS and activating lipogenic transcription factors such as SREBP1c, its reduction suggests a protective effect. Similarly, SREBP2, which regulates de novo lipogenesis, was upregulated in ethanol-treated cells but downregulated after the AgNPs treatment. Ethanol-induced oxidative stress also led to the suppression of antioxidant markers such as Nrf2 and HO-1, both of which were restored upon AgNPs co-treatment, indicating a re-establishment of the redox balance. Furthermore, ethanol triggered an increase in pro-inflammatory cytokines and a concurrent decrease in the anti-inflammatory cytokine IL-10. The AgNPs treatment reversed this trend, enhancing IL-10 expression and supporting the restoration of anti-inflammatory homeostasis (Figure 9A).

Given that colonic inflammation and increased intestinal permeability exacerbate liver damage in in vivo conditions, we evaluated the expression of tight-junction proteins, including ZO-1 and claudin, in colonic cells. Ethanol-treated cells exhibited significant downregulation of these proteins, consistent with previous reports [20,21], indicating impaired barrier integrity. However, co-treatment with AgNPs restored their expression, demonstrating a protective effect on epithelial integrity. Additionally, the expression of pro-inflammatory cytokines TNF-α and IL-6 was reduced in the treatment group, suggesting attenuation of ethanol-induced inflammation (Figure 9B). These findings highlight that, even at low concentrations, AgNPs can mitigate ethanol-induced damage and help preserve intestinal barrier function.

## 4. Discussion

Among the various types of nanoparticles, AgNPs have been extensively studied for their potent antimicrobial and therapeutic properties. In the green synthesis approach, plant-derived bioactive compounds facilitate the reduction of silver ions (Ag^+^) to elemental silver (Ag^0^). This reduction process is primarily driven by naturally occurring phytochemicals, such as tannins, carboxylic acids, aldehydes, and phenolic groups, which act as both reducing and stabilizing (capping) agents [22]. The formation of green AgNPs depends on environmental factors during synthesis, including the concentration of AgNO_3_, pH, temperature, and incubation time. Under these parameters, plant metabolites promote the formation of uniformly shaped nanoparticles [23]. Aqueous plant extracts provide a suitable environment for the dissociation of ionic compounds present in plant material, and, furthermore, assist stable nanoparticle synthesis [24,25]. Due to their multifunctional properties, green-synthesized AgNPs have been explored as potential agents with antimicrobial [26], anti-inflammatory, antioxidant, and anti-diabetic [27] effects.

Further characterization of nanoparticles is essential to determine their size, shape, and physicochemical properties. In this study, green-synthesized AgNPs were characterized using UV–Visible spectroscopy, X-ray diffraction (XRD), Fourier-transform infrared spectroscopy (FTIR), and scanning electron microscopy (SEM/FE-SEM). UV–Visible spectral analysis revealed a distinct absorption peak at 445 nm, which falls within the typical range of 400–500 nm for AgNPs and corresponds to the surface plasmon resonance (SPR) phenomenon, confirming the formation of AgNPs [28]. SPR arises from the collective oscillation of free electrons on the surface of metal nanoparticles when excited by light. The strong, narrow peak indicated isotropic, relatively uniform nanoparticles, whereas broader peaks at higher wavelengths are characteristic of larger particles [29,30]. Along with that, the XRD data also suggest that the 2θ values correspond to silver lattice planes. A comparative analysis was facilitated by referencing the obtained data against the Joint Committee on Powder Diffraction Standards (JCPDS) database, specifically, file number 04-0783, as documented by [14]. Additionally, two supplementary peaks, discernible with asterisks at 27.64° and 31.91°, potentially signify the presence of additional compounds within the plant extract, eliciting intense peak responses [31]. Furthermore, the FTIR spectra exhibited the same peaks as those of the plant extract (see Supplementary Materials), indicating that the particles possess similar characteristics to those of the plant extract. We also observed that the peaks from plant extracts were much sharper than those obtained from nanoparticles. As the particles are formed during the reduction process, the OH banding is observed at 3448 cm^−1^, which is attributed to the presence of the OH group and -C=O activity. Green nanoparticle synthesis is based on the oxidation and reduction process of extracted molecules and metal solutions. Here, the presence of the OH group may facilitate the reduction process that forms the nanoparticles [28,32,33]. The peaks at 2924 and 2825 cm^−1^ indicate the -C-H stretching due to phytoconstituents such as phytochemicals, tannins, and flavonoids [34,35]. Peaks near 1380 and 1039 cm^−1^ correspond to -C-N- vibrations, showing the presence of aliphatic and amine groups [36]. The presence of aromatic -C-C-, -C-H-, and -C-O- corresponds to 1608, 1438, and 1250 cm^−1^, respectively. The -C-H- stretching at 2924 and 2854 cm^−1^ is due to the presence of asymmetric methylene, which is prominent in plant extracts [37]. A stretch bending at 1329 cm^−1^ corresponds to the -N-H- amide linkage, which is also present in the plant extract [28]. The peak at 548–900 cm^−1^ is attributed to aromatic group and alkyl halides, suggesting the presence of bioactive compounds on nanoparticles [38]. 

Morphological examinations were conducted using SEM and FE-SEM, which revealed that the nanoparticles were spherical in nature and had an average size of approximately 110 ± 20 nm. The size was confirmed using DLS. The DLS data showed a peak at 155.8 d.nm, and the z-average size was 119.5 d.nm. The hydrodynamic size of the nanoparticles may be influenced by the phytochemicals present in the leaf extract. To minimize background scattering during analysis, the nanoparticles were properly diluted [39]. The single peak obtained indicates that the sample exhibits size uniformity, suggesting that the particles are not aggregated. As DLS measures the hydrodynamic size of nanoparticles and the microscopic method measures the core size of nanoparticles in dehydrated form, the particle size would be different in both approaches [40]. This was also observed in Ghasemi’s research [26]. The PdI is 0.22 which states that the sample is monodispersed and uniform throughout the solution; moreover, particles with sizes below 150 nm and a PdI close to 0.3 are generally considered suitable for efficient cellular uptake [41].

Various nanoparticles are synthesized via a green method, showing hepatoprotective effects [19,42]. Excessive ethanol exposure leads to significant liver injury, primarily by damaging hepatocytes through the induction of pro-inflammatory cytokines. This process is initiated by the activation of cytochrome P450 2E1 (CYP2E1), a key enzyme responsible for ethanol metabolism and a major contributor to ethanol-induced hepatotoxicity [43]. CYP2E1 activation leads to the generation of ROS, which in turn causes oxidative stress, resulting in lipid peroxidation, DNA damage, and eventual cell death [44]. The liver’s capacity to counteract this oxidative damage through antioxidant defenses becomes insufficient under chronic ethanol exposure, leading to a disruption in redox homeostasis. Consequently, as the central organ for ethanol metabolism, the liver sustains substantial damage and loses its ability to maintain physiological balance [45]. In this context, green-synthesized nanoparticles showed a positive response, suggesting their potential utility in mitigating ethanol-induced liver damage.

Here, a cell viability study was conducted under both conditions. The cells were treated with different concentrations of AgNPs, and, in addition, 0.2 M ethanol media was provided to the cells. Cells with higher concentrations of AgNPs exhibited higher cell death, whereas those with lower concentrations showed better cell viability, even after exposure to ethanol. This effect may be attributed to the size-dependent properties of the nanoparticles. Smaller particles have a higher surface area-to-volume ratio, promoting greater cellular uptake and interaction, which increases cytotoxicity at elevated doses. In contrast, larger nanoparticles are less readily internalized, limiting their biological activity. Prior studies have shown that smaller AgNPs can diffuse across the cell membrane more effectively, whereas larger particles may enter via ion channels [46,47]. Consequently, cell viability tends to decrease with increasing nanoparticle dosage. In the present study, green-synthesized AgNPs demonstrated protective effects against ethanol-induced cytotoxicity at lower concentrations, promoting cell survival and proliferation.

Along with cancerous cells, the viability of AgNPs was also assessed in a normal rat liver cell line (BRL3A). These normal cells differ from cancerous cells in terms of membrane permeability, genetic stability, and proliferation characteristics [48]. For this reason, normal cells exhibited higher viability even at elevated concentrations of AgNP exposure compared to cancerous cells. Since cancer cells inherently produce higher levels of ROS [49], increasing the concentration of AgNPs further enhanced ROS generation in combination with ethanol, leading to increased oxidative stress and subsequent cell death. Previous studies have also reported that high concentrations of AgNPs can induce G1 and G2 cell cycle arrest, ultimately resulting in cytotoxicity [50,51].

- Anti-inflammatory mechanism:

Molecules generated from oxidative stress alter cellular and nuclear morphology by increasing membrane permeability and promoting pro-inflammatory cytokines, such as TNF-α, a key component of necrotic pathways [12,52]. In parallel, pro-inflammatory cytokines generated in colonic epithelial cells can also suppress the anti-inflammatory cytokines such as IL10. These mediators can translocate to the liver via the highly interconnected gut–liver axis, thereby exacerbating hepatic injury [53,54].

In this study, treatment with green-synthesized AgNPs appeared to mitigate cellular damage and enhance viability, resulting in a nuclear morphology comparable to that of untreated control cells (Figure 6) and potentially interrupting the pathological cascade associated with ethanol exposure (Figure 5). It enhances the cell viability and mitigates the nuclear damage. Larger-sized AgNPs synthesized via green methods are generally unable to penetrate the nuclear membrane, thereby reducing the risk of interfering with nuclear processes. These nanoparticles have been reported to preserve DNA integrity even at relatively high concentrations. Studies have shown that AgNPs derived from plant extracts support genomic stability and may aid in maintaining nuclear architecture [55,56], thus offering therapeutic potential in disease amelioration [57]. By preserving nuclear integrity, these nanoparticles may help sustain a healthier cellular environment, reducing the expression of pro-inflammatory cytokines and promoting the production of anti-inflammatory and antioxidant molecules in liver and colonic cells.

Green AgNPs have shown promising potential in wound healing by exhibiting anti-necrotic activity, promoting cell proliferation, and enhancing cell migration, all of which contribute to improved cell survival and disease amelioration [58]. *Enicostemma littorale* plant extract has been reported to exert protective effects against necrosis by downregulating TNF-α expression and facilitating tissue regeneration [59,60]. As the particles are synthesized on a nanoscale and with similar plant extract, it is possible that they retain similar properties to the plant itself but exhibit increased bioavailability and enhanced bioactive effects compared to the crude extract. In addition to their biocompatibility, green-synthesized AgNPs possess intrinsic antioxidant properties, contributing to their cytoprotective effects.

- Antioxidant mechanism:

Green-synthesized nanoparticles exhibit intrinsic antioxidant activity, effectively inhibiting the formation of ROS such as hydrogen peroxide (H_2_O_2_), superoxide anion (O_2_^−^), and hydroxyl radicals (•OH). These ROS can interact with cellular macromolecules, including DNA, proteins, and lipids, leading to structural modifications and functional impairments that contribute to cellular damage and apoptosis. As mentioned previously, due to ethanol metabolism, the primary antioxidant defense mechanism is suppressed, along with increased expression of proinflammatory cytokines, resulting in more cellular damage. The generated ROS not only triggers lipid peroxidation but also impairs endogenous antioxidant systems by diminishing the protective activity of nuclear factor erythroid 2-related factor 2 (Nrf2) and heme oxygenase-1 (HO-1) [61].

In this study, elevated levels of ROS due to ethanol further decreased due to the AgNPs treatment, showing the antioxidant activity of nanoparticles (Figure 7). Compared to chemically synthesized AgNPs, green-synthesized variants exhibit an enhanced antioxidant capacity, which is attributed to their ability to effectively donate or accept electrons, thereby stabilizing free radicals and mitigating oxidative damage [62,63]. This electron transfer ability enables them to quench ROS and maintain cellular redox homeostasis efficiently.

- Antilipidemic activity:

Ethanol exposure predominantly affects lipid metabolism by altering the expression of key regulatory genes such as peroxisome proliferator-activated receptor alpha (PPAR-α) and sterol regulatory-element-binding protein 1c (SREBP1c). Under ethanol-induced stress, PPAR-α, a gene responsible for promoting lipid catabolism, becomes downregulated, while SREBP1c is upregulated, which promotes lipogenesis. Additionally, ethanol promotes de novo lipogenesis by inhibiting β-oxidation, resulting in intracellular lipid accumulation. This causes hepatic fat accumulation and steatosis [64]. Moreover, the impact of ethanol is not limited to hepatic tissue; colonic epithelial cells are also adversely affected, partly due to lipid accumulation. Several studies have indicated that ethanol induces the formation of lipid species such as ceramides and hexosylceramides, which, when elevated, can disrupt cellular function, contribute to insulin resistance, and exacerbate inflammation [65,66]. These alterations further impair tissue homeostasis and are associated with the progression of metabolic and inflammatory disorders. As the green-synthesized AgNPs treatment was administered to ethanol-treated cells, it exhibited better lipolysis than cells treated with ethanol alone (Figure 8). The antilipidemic activity exhibited by the green-synthesized AgNPs may be attributed to the presence of swertiamarin, a bioactive flavonoid found in the *Enicostemma littorale* plant extract used for nanoparticle synthesis. Notably, swertiamarin has been reported to possess hepatoprotective and lipid-regulating properties, primarily through the modulation of cholesterol metabolism and the enhancement of lipolysis. Thus, the observed lipid-lowering effects of the AgNPs may result from the combined physicochemical properties of the nanoparticles and the bioactivity conferred by phytochemicals such as swertiamarin [67,68]. However, given the low water solubility of swertiamarin and the limited solubility of certain other compounds in aqueous extracts, it remains unclear whether the antilipidemic activity can be attributed to any specific constituent of the plant extract. Furthermore, researchers demonstrated the hypolipidemic effect of plant aqueous extract against ethanol-induced damage, decreasing cholesterol and triglyceride levels in serum [69,70,71].

- Gut tight junction protein restoration:

Beyond hepatic effects, ethanol also compromises intestinal barrier integrity by disrupting tight-junction proteins such as ZO-1, occludin, and claudin. This disruption is partly mediated by increased tyrosine kinase activity, which downregulates ZO-1 expression and impairs the functionality of tight junctions. Ethanol also inhibits hypoxia-inducible factor 1-alpha (HIF-1α), leading to epithelial cell injury in the colon [72]. The resulting epithelial damage elevates local inflammation, promoting the release of pro-inflammatory interleukins, which further deteriorate tight-junction integrity and exacerbate intestinal permeability, contributing to systemic inflammation. Green-synthesized AgNPs effectively preserved intestinal barrier integrity and tight-junction protein expression, thereby reducing colonic inflammation.

Since ethanol disrupts both hepatic and intestinal homeostasis, it is essential to target the underlying mechanisms in both organs. Given the gut’s secondary yet significant role in disease progression, a therapeutic strategy that addresses both hepatic and intestinal cellular dysfunctions is crucial for effective interventions.

## 5. Conclusions

**Figure 10:**
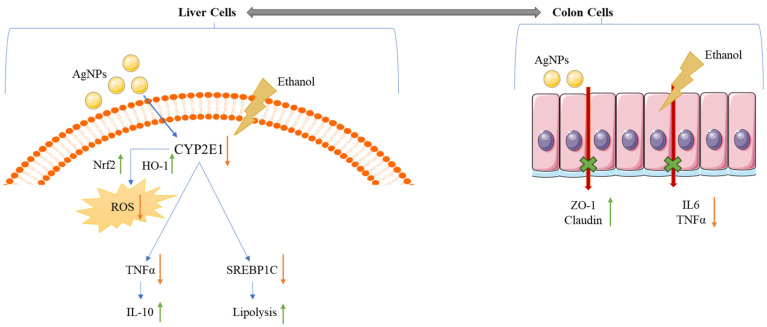
The liver and colon are interconnected via the portal vein, enabling ethanol-induced pro-inflammatory cytokines and toxins from the colon to reach the liver. Ethanol also directly increases hepatic ROS and inflammation and disrupts lipid metabolism. Green-synthesized AgNPs help reverse both primary liver injury and secondary gut-derived inflammation.

Ethanol-induced liver damage is a significant global health concern, contributing to the high mortality rate among liver-related disorders. It is primarily a lifestyle-associated disease caused by excessive alcohol consumption, which not only impairs liver function but also disrupts gut homeostasis by damaging tight-junction proteins and triggering intestinal inflammation. This breakdown in gut barrier integrity facilitates the translocation of pro-inflammatory cytokines and toxic metabolites to the liver via the portal circulation, thereby accelerating disease progression. To address this, green-synthesized nanoparticles have emerged as promising therapeutic agents, particularly because many plant-derived compounds suffer from poor water solubility and limited intestinal absorption. Nanoparticle formulation enhances their bioavailability and therapeutic efficacy. In this study, green-synthesized AgNPs derived from *Enicostemma littorale* extract demonstrated potent antioxidant, anti-inflammatory, anti-lipidemic, and cell-proliferative activities in both hepatic and colonic cell models (Figure 10). In smaller amounts, the particles tend to decrease the inflammation caused by ethanol exposure and help to rejuvenate tight junctions. These properties of AgNPs tend to help mitigate ethanol damage and alleviate the disease. At higher concentrations, AgNPs exhibit a lethal effect on both cell lines. These properties collectively contribute to mitigating the damage caused by ethanol. For future studies, in vivo validation is recommended to authenticate the protective effects of green-synthesized AgNPs and to explore their potential as a therapeutic strategy for alcohol-associated liver and gut dysfunction.

## Figures and Tables

**Table 1:** Human primer sequence list.

Gene Name	Forward Primer (5′-3′)	Reverse Primer (5′-3’)
*18s*	GATGGTAGTCGCCGTGCC	GCCTGCTGCCTTCTTGG
*TNF-α*	CTCTTCTGCCTGCTGCACTTG	ATGGGCTACAGCTTGTCACTC
*ZO-1*	TATTATGGCACATCAGCACG	TGGGCAAACAGACCAAGC
*Claudin-1*	CCATCAATGCCAGGTACGAAT	TTGGTGTTGGGTAAGAGGTTGTT
*IL6*	CATCCTCGACGGCATCTCAG	GCAGAAGAGAGCCAACCAAC
*IL10*	ACTGCTAACCGACTCCTTA	TAAGGAGTCGGTTAGCAGT
*NrF2*	GAGAGCCCAGTCTTCATTGC	TGCTCAATGTCCTGTTGCAT
*CYP2E1*	AACTGTCCCCGGGACCTC	GCGCTCTGCACTGTGCTTT
*SREBP2*	CTCCATTGACTCTGAGCCAGGA	GAATCCGTGAGCGGTCTACCAT

**Table 2:** Determination of functional group.

Peak Wavelength (cm^−1^)	Functional Group Assigned
3448	OH group presence
2924, 2825, and 1438	-C-H- stretching showing presence of phytochemicals
1380 and 1039	-C-N- group vibration indicating presence of aliphatic and amine group
1329	-N-H- group, amine linkage

## Data Availability

The data presented in this study are available on request from the corresponding author.

## Supplementary Materials

The following supporting information can be downloaded at: https://www.mdpi.com/article/10.3390/pharmaceutics17070895/s1, Figure S1: FTIR peaks of plant extract.; Figure S2: EDS spectra of synthesised nanoparticle.
