# Supplementary material for: Silver Nanoparticles Synthesized from Enicostemma littorale Exhibit Gut Tight Junction Restoration and Hepatoprotective Activity via Regulation of the Inflammatory Pathway
Source: Pharmaceutics. 2025 Jul 9;17(7):895. doi: 10.3390/pharmaceutics17070895 (PMC12298411; doi:10.3390/pharmaceutics17070895)
Supplement: Supplementary file 1 [file pharmaceutics-17-00895-s001.zip › pharmaceutics-3719496-supplementary.pdf]

FTIR data of plant extract

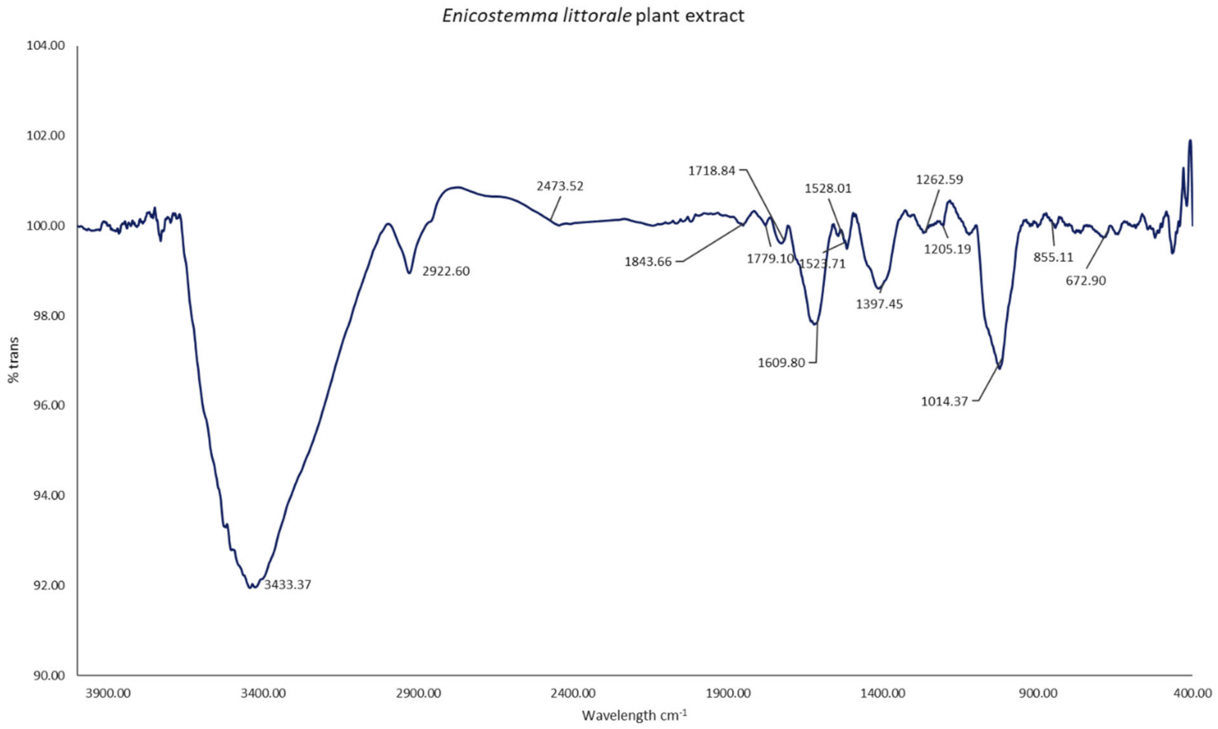

Supplementary Figure S1. FTIR peaks of plant extract.

EDS:

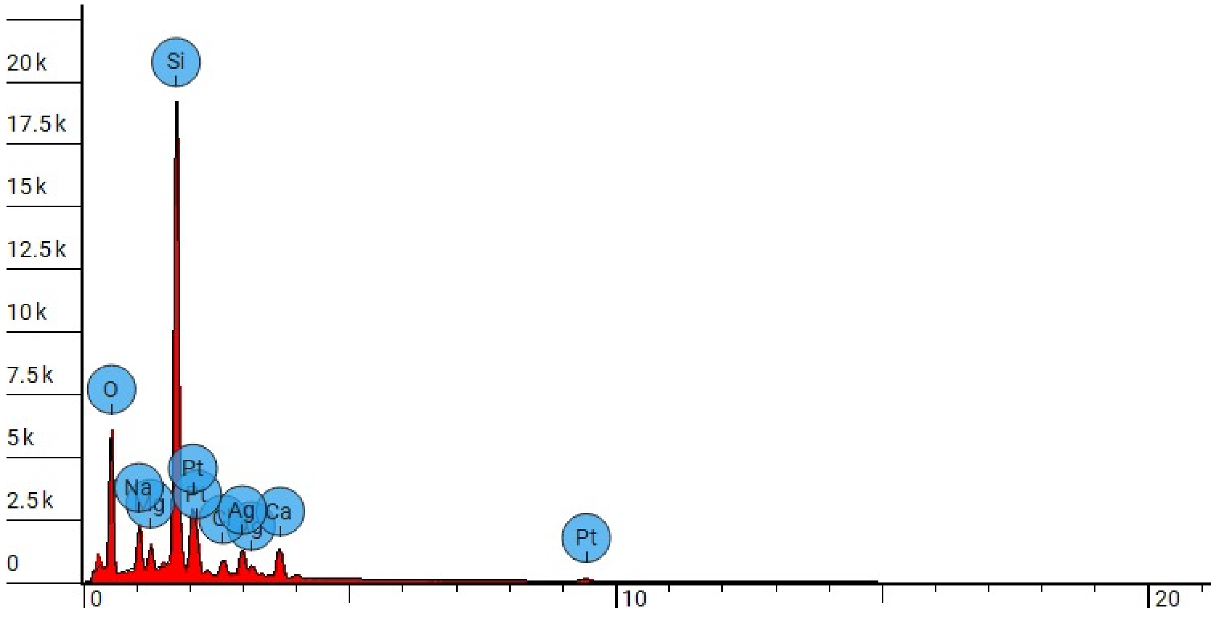

Supplementary Figure S2. EDS spectra of synthesised nanoparticle.

Si and Pt shows due to the drop cast of sample was done on glass cover slide and further sample was coated with platinum. So, the Si and Pt peaks were observed. But other than that the peaks observed are from plant extract and $AgNo_3$ solution.
